# Smart Home Advancements for Health Care and Beyond: Systematic Review of Two Decades of User-Centric Innovation

**DOI:** 10.2196/62793

**Published:** 2025-05-20

**Authors:** Youna Park, Jieun Han

**Affiliations:** 1 Graduate School of Technology and Innovation Management Hanyang University Seoul Republic of Korea

**Keywords:** smart home, systematic literature review, thematic analysis, health care, user needs, ambient intelligent home, user centric innovation

## Abstract

**Background:**

The rapid advancement of Internet of Things and artificial intelligence technologies has driven significant growth in the demand for smart home products, with household penetration projected to increase from 77.6% in 2025 to 92.5% in 2029. Despite this growth, much of the existing research adopts a technology-push approach, focusing primarily on user adoption and acceptance from the perspective of technology providers rather than addressing the evolving needs and experiences of users.

**Objective:**

This study seeks to bridge this gap by conducting a comprehensive review of 20 years of smart home literature (2000-2023) using bibliometric and thematic analysis. The objective is to align future research with user needs and market trends by examining technology, user behavior, and service perspectives to inform academic and industry developments.

**Methods:**

The analysis was carried out in four steps: (1) collecting 5981 abstracts from the Web of Science database (from 2000 to the first half of 2023), (2) preprocessing and refining the data, (3) applying topic modeling to 1003 selected papers, and (4) combining bibliometric network analysis with qualitative, in-depth interpretation.

**Results:**

This study identified 110 key topics across 4 distinct periods, providing insights into 2 decades of advancements in technology, applications, and user-centered research. The findings illustrate the progression of smart home technologies from wireless networks to Internet of Things and artificial intelligence, the expansion of services from basic monitoring to applications such as energy management and health care, and a shift in user profiles from early adopters to underserved populations. The study highlights the limitations of the technology-push approach and underscores the importance of understanding diverse user groups and improving user experiences. It also emphasizes the potential of ambient intelligence environments to intuitively adapt to the health and lifestyle needs of all residents, simplifying and enriching daily life.

**Conclusions:**

This research delivers valuable academic and practical insights into the evolution of the smart home domain, showcasing a shift from technology-push strategies to user-centered approaches. By prioritizing user satisfaction and quality of life—especially in health care—the findings open new commercial opportunities and pave the way for innovative, real-world smart home solutions tailored to contemporary user needs.

## Introduction

### Changing Concepts of Smart Home Require New Analytics to Uncover Insights

The smart home industry, fueled by core technologies of the Fourth Industrial Revolution such as artificial intelligence (AI) and the Internet of Things (IoT), has attracted significant attention, permeating various products, services, and platforms. Over the past 2 decades, particularly since 2010, smart homes have witnessed substantial advancements in both industry and research. Initially developed for home automation in the early 2000s, the industry has evolved significantly with the emergence of wireless internet, the widespread adoption of smartphones, the proliferation of IoT devices, and advancements in AI. These developments have propelled smart home research and applications into a phase of rapid growth, marking the beginning of a new era of innovation and integration.

Given this context, several experimental studies have been conducted, including the Aware Home at the Georgia Institute of Technology, PlaceLab at the Massachusetts Institute of Technology, R-House at Indiana University, Easy Living at Microsoft Research, the UKDRI Smart Home at Imperial College London, and Keio The Smart Space at Uiku University in Japan [1,2]. These seminal studies have played a crucial role in advancing our understanding of smart home technologies and their implications. By delving into user behaviors, preferences, and acceptance of smart home technologies in real-world settings, these studies have provided useful insights [3]. The findings from these studies have not only informed the development of user-centric smart home solutions but also shaped the trajectory of future research and innovation in the field. By addressing user needs and preferences, researchers and industry practitioners can design more intuitive and effective smart home technologies that enhance the overall user experience and promote widespread adoption.

Therefore, this study aims to synthesize the conceptual framework and scholarly research related to smart homes over the past 2 decades, while also examining future research directions. To achieve this objective, the methodology uses bibliometric analysis and thematic analysis. These methodologies identify and classify smart home literature across a temporal spectrum, facilitating a comprehensive understanding of the field’s evolution.

This study compiled 5981 papers published between 2000 and 2023 from the Web of Science database, using search terms associated with smart homes and users. Subsequently, a systematic literature review (SLR) was undertaken, followed by topic modeling on 1003 papers.

Furthermore, 42 selected papers underwent in-depth analysis through literature review. This research scrutinized the time series change process, yielding comprehensive insights into smart homes. Its significance lies in its capacity to explore various facets, including smart home products, services, and user dynamics, thereby facilitating adaptability to future changes. This study aims to address the following research questions (RQs):

- RQ1. What has been the trend in smart home research over the past 20 years?
- RQ2. In which domains has smart home research been primarily conducted?
- RQ3. What is the optimal framework for effectively conducting smart home research?
- RQ4. What are the main topics discussed in the diachronic analysis of smart home research over the past 20 years?
- RQ5. How has smart home research evolved over different periods?
- RQ6. What are the key challenges and opportunities in the development of smart home technologies?

### Changing the Concept of a Smart Home

The concept of the “smart home” traces back to 1975, when it was defined as a residential system equipped with interactive automation technology, empowering residents to manage various home functions such as lighting, temperature control, multimedia utilization, and security monitoring. The actual term was coined by the American Home Builders Association in 1984, when the concept gained momentum. In the early 20th century, the advent of electronic technologies such as vacuum cleaners, food processors, and washing machines marked a significant stride toward enhancing convenience and comfort within households [1,4]. The commercialization of the first home automation platform, X10, in 1970 represented a milestone, transmitting digital data via radio frequencies through existing electrical wiring [5]. The late 1990s witnessed a surge in home network development opportunities, propelled by the widespread adoption of high-speed internet. The emergence of the IoT has significantly changed the concept of smart homes. In the late 2000s, the adoption of smartphones increased the popularity of smart homes through mobile apps. Throughout its evolution, smart homes have gone by various names, such as home networks, digital homes, home automation, and intelligent homes, reflecting the expanding scope and definition of the concept [6].

In the industrial sector, major technology companies, including Google, Amazon, Apple, Samsung, Huawei, among others, have introduced various smart devices and home automation systems [1]. The advent of integrated home automation solutions has significantly expanded the scope of smart homes. Since 2010, the proliferation of voice-controlled AI speakers such as Amazon Echo and Google Home has been remarkable. These devices have expanded beyond their initial function of controlling home appliances through voice recognition, evolving into comprehensive platforms offering a wide array of services. These services range from accessing music content to facilitating web-based shopping [7].

While the traditional concept of the smart home primarily focused on providing convenience and comfort, the modern smart home landscape, particularly from 2018 onward, has shown its potential to deliver practical services aimed at enhancing quality of life. These services encompass aspects such as security, energy saving, and telehealth underscoring the evolving nature of smart home technology toward more holistic and user-centric functionalities [8].

In the early stages of smart home development, the primary focus revolved around integrating products and services through communication technology. The goal was efficiency, with this integration aimed at simplifying user control and management, ultimately saving time. While there was some discussion regarding the necessity for services to align with user needs, the predominant focus of research and development centered on early adopters exploring products or services with new technologies. These experiments were conducted by constructing and evaluating specific environments, albeit often detached from the authentic user experience.

In recent years, significant strides in IoT and sensor technology have resulted in transformative changes. These technologies empower devices to detect electricity and water usage, learn from user behaviors, and autonomously adapt. Consequently, personalized services have emerged, capable of tailoring energy usage to users’ lifestyles, thereby facilitating energy conservation. This evolution marks a notable shift in the smart home sector, evolving beyond efficiency to enhance users’ quality of life, viewed through the 3 perspectives of technology, services, and user dynamics.

### An Analytical Framework for Insights Into Smart Home

Although the smart home industry is diversified and has gradually entered the mainstream market, research remains predominantly skewed toward the technological perspective, often overlooking the broader perspectives of services and user dynamics [9].

Existing studies tend to concentrate on technical aspects such as device capabilities, infrastructure and architecture development, and applications, thereby leading some scholars to call for a more user-centric approach that prioritizes the actual benefits of smart homes technology [10]. From a technology perspective, research on smart homes typically revolves around the integration of various technologies, including home automation, automatic control systems, communication networks, connected devices and services, remote access and control, and home intelligence. Conversely, the service perspective primarily focuses on control, monitoring, and response support for technology management, with additional exploration into various services such as telehealth services for promoting healthy living [11].

Research from the user perspective in smart homes has primarily focused on understanding adoption and utilization patterns, emphasizing adoption factors, challenges, barriers, and postadoption usability evaluations. According to the findings by Wilson et al [12], the adoption rate of smart home systems remains low, potentially due to a mismatch between developed products and users’ actual needs. This highlights the pressing need to account for user diversity, particularly aging populations. The evolution of smart home health care has shifted chronic disease management from hospitals to home environments, realizing concepts such as “aging in place.”

Advances in mobile technologies, including IoT and sensors, have greatly enhanced usability and personalized care [13]. These innovations accelerate adoption cycles by enabling remote health management and fostering personalized, user-focused smart home health care solutions, leveraging technologies such as AI and blockchain. This transition underscores the importance of user-centered design and privacy-conscious policies while addressing ethical and legal challenges in health care.

In this study, we will conduct research from the following 3 perspectives. Conducting a macro-level study encompassing technologies, services, and user dynamics will enable us to explore user needs and identify gaps between technologies and services in future smart homes. This holistic approach is crucial for bridging the divide and ensuring alignment between technological advancements and user expectations.

### SLR and Thematic Analysis

Systematic quantitative analysis methods are useful tools for scientific evaluation, enabling the identification of contributions and impacts made by pioneers and practitioners in a field. These methods also facilitate the examination of underlying influences and afford a macro view of the existing literature [14]. By using scientific methodologies and systematic quantitative analysis, researchers can gain comprehensive insights into the landscape of a particular domain, thereby advancing knowledge and understanding within the field. SLR centered on thematic and content analysis using tools, such as Leximancer (Leximancer Pty Ltd), has been applied across a wide range of disciplines. Recent studies have used this method to conduct comprehensive literature reviews in diverse areas including digital banking, e-market publishing, cross-cultural psychology, small- and medium-sized enterprises, and studies on entrepreneurship evolution.

These research methods enable researchers to systematically observe and analyze changes and continuities in thematic interests over time, mitigating distortion caused by fluctuations in the volume of literature. Such analyses provide insights into the emergence, evolution, and eventual disappearance of certain concepts, providing a nuanced understanding of the topic under investigation [15].

As a result, this approach contributes to a more foundational and contextualized interpretation of trends and opportunities within the field of study, thereby enriching academic and practical knowledge. Moreover, by comparing Prominence Index values using this method, discrepancies between periods across different time frames can be readily discerned, facilitating a more refined interpretation of these disparities. This allows for a deeper exploration of temporal variations and enhances the overall interpretative capability of the analysis.

In a study investigating the evolution of business-to-business (B2B) marketing topics, researchers analyzed 328 papers from B2B marketing journals from 1993 to 2014. The scientific analysis revealed 4 themes and 86 concepts, categorized across 4 distinct time periods. Notably, a consistent theme throughout the various time frames underscored the significance of relationships in B2B marketing. Using Prominence Index analysis, the study elucidated the evolution of these concepts over time, highlighting changes and differences between time periods [15].

Another literature review traced 3 decades of research within the field, using Leximancer to conduct content analysis. The study analyzed 211 papers from the journal “Electronic Markets,” along with 356 presentations from the Bled eConference. The analysis identified 13 main themes, with notable overlaps including information, services, business, online, social, and systems. The study detailed the similarities and discrepancies between these identified themes, providing a thorough assessment of their impact on the field [16].

In addition, a literature review exploring the application of AI in the banking sector since 2005 conducted a systematic analysis of 44 papers. Thematic and content analysis using Leximancer uncovered several pivotal themes, leading to the proposal of an AI banking services framework designed to reconcile the disparity between research and industry practices. The analysis identified strategy, process, and customer relationships as key areas of research, providing insights into strategic planning and value optimization for the future use of AI technology within banking operations [17].

As shown in previous research, a systematic review offers a comprehensive understanding of a subject matter, revealing overarching insights and subtle changes and trends within the field. Building upon this premise, this study aims to analyze a wide range of literature pertaining to smart homes spanning the last 2 decades. The primary objective of this study is to discern evolving characteristics across different time periods, thereby furnishing a macro view from 3 distinct perspectives.

To achieve its goals, the study adopts a hybrid approach that combines qualitative and quantitative research methods, using Leximancer. This integrated methodology not only facilitates a nuanced comprehension of smart home technologies but also allows for a deeper exploration of their evolution over time. Bibliometric analysis provides quantitative insights into publishing trends, citations, and collaborations, thereby improving understanding of the main themes and gaps in the academic environment. Conducting an SLR guided by the PRISMA (Preferred Reporting Items for Systematic reviews and Meta-Analyses) framework, the study addresses key research questions in a structured manner (Multimedia Appendix 1). By synthesizing these qualitative, quantitative, and bibliometric insights, the research aims to significantly contribute to the existing body of knowledge on smart home technologies and their development.

## Methods

### Dataset Collection

For data collection, we used the Web of Science platform because of its credibility and extensive coverage of peer-reviewed papers across diverse disciplines [18]. Leveraging the broad scope of this database, we conducted a thorough search and filtering process to ensure inclusivity and breadth in our literature selection. Our aim was to compile a comprehensive collection of literature on smart homes by combining various types of literature [19]. Aligned with our research framework, we structured our queries as follows: Query = (“smart home” OR “smart home user”) AND (“behavior,” “acceptance,” “adoption,” “perspective,” “learning,” “use,” “evaluate”).

### Data Preprocessing and Refinement

A rigorous manual review process was used, involving scrutiny of titles and abstracts, content and quality assessment, and backward and forward searches. This process was used to select papers for comprehensive content analysis.

The initial stage involved a passive review based on titles and abstracts. Subsequently, these papers underwent a meticulous screening process, wherein abstracts were thoroughly examined, and promising ones were further scrutinized by reviewing relevant sections of the full text. These papers were then subjected to topic modeling to identify themes for each time period. During this phase, particular attention was paid to the thematic relevance of each paper, with a focus on encompassing a broad spectrum of smart home technologies, services, and user perspectives. Papers predominantly focused on specific technological aspects were excluded to maintain the study’s comprehensive scope. In addition, studies limited to specific products or lacking generalizable insights about smart products were also omitted from the final selection [20].

The subsequent phase involved a thorough assessment of the remaining papers for their content and relevance. Considering that recent literature often originates from conferences before undergoing peer review for journal publication, special attention was given to conference papers. A subset of papers was selected as an initial sample, including those presented at notable conferences such as *Human Factors in Computing Systems*. To ensure that the literature list was comprehensive and reliable, backward and forward citation searches were conducted, which is a critical step in concept-driven literature reviews [21-23]. This comprehensive approach facilitated the gathering of a diverse range of literature, providing valuable insights into the evolving landscape of smart home research over the past 2 decades.

The selection of these papers was made to maintain proportional representation across different time periods, ensuring a balanced analysis of trends and developments. Exclusion criteria were carefully defined in alignment with the systematic approach inspired by PRISMA guidelines. Papers were excluded if they lacked sufficient methodological rigor, did not align with the research objectives, or focused on unrelated domains. This systematic process ensures the selection of a robust and representative body of literature for analysis.

### Data Analysis

The data analysis was conducted in 3 subanalysis steps. First, we conducted a descriptive analysis based on publication frequency. This allowed us to identify the fields within smart home research and the primary journals contributing to this field.

After the descriptive analysis, we performed topic modeling on the papers for the second time-series analysis. These papers were segmented into time periods to explore the evolution of smart home research over time. To establish the time intervals, we divided the timeline into four 6-year phases, taking into consideration the distribution of publications across the years. To compare the different time periods, we conducted topic analysis through concept mapping using Leximancer.

This software-based analysis allows researchers to overcome the biases and errors inherent in manual methods and avoiding subjective interpretations often associated with handwritten notes. Leximancer automatically identifies key concepts and themes from the dataset, facilitating deeper interpretation. A theme, in this context, represents a set of concepts that are interconnected, rather than simply the most frequently occurring word [24].

The analytical process involved conducting textual content analysis on the abstracts of the literature. Through this process, underlying concepts (common texts identified here by concept names) and themes (clusters of concepts) were determined to ascertain the primary topics within smart homes research for both entire period and each distinct period.

Leximancer is particularly suitable for exploratory studies aiming to develop comprehensive conceptual models. It operates on Bayesian probability theory to select reliable and reproducible concepts and topics (or clusters of topics), thereby predicting trends within a system without introducing bias from preconceived ideas. The software facilitates a quantitative approach to qualitative research and yields an overall conceptual structure with detailed insights into specific themes within the smart home literature over time through analysis [25].

Visual concept maps and statistical output, created using Leximancer, were used to identify key concepts. Through the creation of a visual concept map, complemented by statistical output, we determined the main themes found in the textual data, highlighted the interrelationships among themes, and indicated which files from which time periods can be characterized by particular themes or topics [26]. This approach proved invaluable for uncovering key themes and concepts within the text data, offering a graphical representation (ie, concept maps) for analyzing clusters of text data and exploring deeper contextual connections as the text was mined [27]. The rationale behind using text mining analysis with Leximancer is to visually chart the conceptual structure of smart homes, interpret thematic emphases and their interrelations, and reveal the interdependencies and proximities among themes across each time period, thereby enhancing comprehension and interpretation of the past and potential future [28].

Third, we conducted a qualitative in-depth analysis, specifically content analysis, to classify the topics identified according to their respective time periods. This qualitative scrutiny enabled us to extract nuanced insights and provide a contextual understanding of the emerged topics. The results of this qualitative analysis served as a foundation for interpreting the outcomes of the thematic analysis.

## Results

### Data Collection, Preprocessing, and Refinement

**Figure 1:**
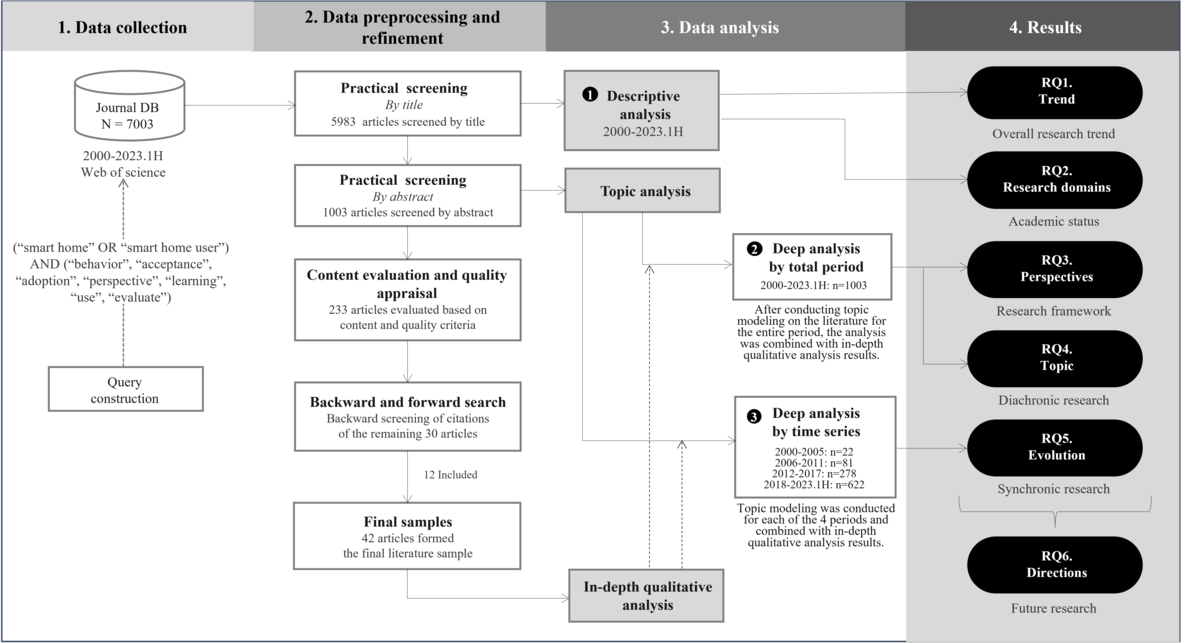
PRISMA (Preferred Reporting Items for Systematic Reviews and Meta-Analyses) flow diagram. The research methodology is quantitative and qualitative analysis through systematic literature review and thematic analysis. 1H: first half; RQ: research question.

Figure 1 shows the overall flow of the research methodology. Using the final search string, we retrieved 7003 papers. After excluding 4 papers published before 2000 and 1016 duplicates, 5983 remained for screening. We excluded 4 documents before 1998 due to their limited representation, which could hinder meaningful analysis. In addition, the low number of papers before 2000 made them unsuitable for inclusion in the 6-year incremental analysis. In the second step, we evaluated the content and quality of these papers. In the third step, we analyzed the 1003 screened papers using Leximancer, a software that uses statistical algorithms to extract topics from text data by analyzing word frequency and co-occurrence patterns. The remaining 233 papers were evaluated based on content and quality criteria. Afterward, 30 papers were selected for backward and forward citation searches. Through this process, 12 additional papers were identified, resulting in a final list of 42 papers [2,6-9,12,14,23,29-62] for in-depth analysis and data extraction.

### Descriptive Analysis and Analysis of Data Over Time

The periodic distribution of research and journal frequency data concerning smart homes provided insights into the trends in research popularity and the primary research areas. It shows a gradual increase in the number of published papers on the topic since 2000, with a sharp increase of more than 2-fold per year observed since 2014. This acceleration indicates a rapid growth in academic interest and popularity, a trend that has persisted similarly since 2018 (*R*^2^=0.617). The corresponding graph can be found in Multimedia Appendix 2.

The statistical analysis of journal frequency yielded insightful findings, with a total of 3082 journals containing 5981 abstracts. Multimedia Appendix 3 shows the most influential journals in smart home research since 2000. *Sensors*, a multidisciplinary journal focusing on sensor technologies, leads with 232 publications, followed by *IEEE Access* and *IEEE Internet of Things Journal*, each with more than 100 papers. *Energies*, *Applied Sciences-Basel*, *Journal of Ambient Intelligence and Humanized Computing*, *Sustainability*, and *Electronics* also boast more than 40 papers. Among the search results, the *IEEE Internet of Things Journal* paper “Edge Computing: Vision and Challenges” stands out as the most cited, with 3527 citations, emphasizing various implementation cases of edge computing and future challenges. The analysis results offer insights into the current stage of the discipline, addressing both the first and second research questions by highlighting the trends in smart home research and identifying influential publications.

The research on smart homes has seen a steady increase since 2000, with a sharp rise in publications after 2014, reflecting growing academic interest and technological advancements. From 5981 abstracts in 3082 journals, *Sensors* leads as the most influential multidisciplinary journal with 232 publications, while the *IEEE Internet of Things Journal*’s paper “Edge Computing: Vision and Challenges” remains the most cited with 3527 citations.

### Deep Analysis by Total Period

**Figure 2:**
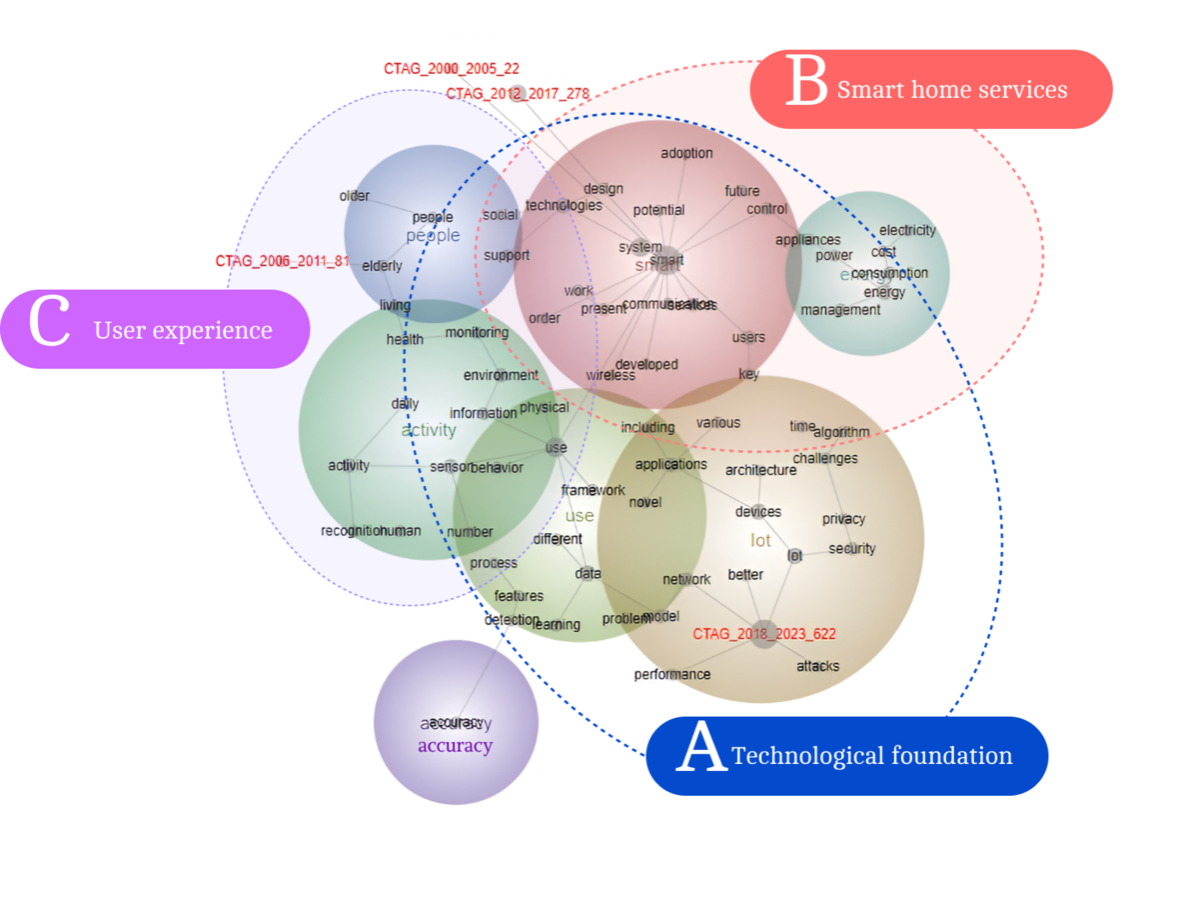
Concept map for the total period (2000 to the first half of 2023).

A total of 7003 papers on smart homes were selected, with 1003 papers specifically used for topic extraction using Leximancer and subsequent in-depth analysis alongside 42 papers from SLR. This process identified 69 concepts grouped into 7 themes, visualized in a concept map “Total Period (2000-1H 2023)” in Figure 2, where larger dots represent more prominent concepts and colored bands indicate thematic groupings—key themes in red and less important ones in purple [63].

Visual concept maps and statistical outputs, including heat maps generated by tools such as Leximancer, were used to analyze and cluster key themes within the text data. These tools grouped concepts based on their frequent co-occurrence, highlighting thematic clusters where key themes are depicted in red and less significant ones in purple [26-28,63]. The resulting heat maps illustrate trends and relationships among topics over different periods, providing insights to trace past research trends and predict future trajectories [24].

The 1003 papers were chosen to represent different periods: 22 (2%) papers from 2000 to 2005, 81 (8%) papers from 2006 to 2011, 278 (28%) papers from 2012 to 2017, and 622 (62%) papers from 2018 to the first half of 2023, reflecting a marked increase in research activity in recent years. The concept map “Total Period (2000-1H 2023)” and red folders labeled “CTAG” represent papers and their alignment with themes over time; the concept map illustrates how the literature from various periods clusters around key themes, indicating trends in smart home research. The 2000-2005 and 2012-2017 periods share similar content, closely linked to the “Smart” and “People” themes. In contrast, the 2006-2011 period aligns more with “People” and “Activity,” while 2012-2017 gravitates toward “Smart” and “Energy.” The latest period, 2018-2023, marks a distinct shift, focusing more on “IoT,” “Use,” “Activity,” and “Energy,” suggesting a broader exploration of smart home applications. “Smart” remains the dominant topic across the periods, followed by “Use” and “IoT,” with each period’s specific focus reflected in the frequency and prioritization of these themes (Table 1).

This study uses a macro-level framework to examine technologies, services, and user dynamics, aiming to explore user needs and bridge gaps between technological advancements and future smart home expectations. The full papers were divided into time periods and analyzed from 3 perspectives based on the results from Leximancer (Figure 2): (A) technological foundation, (B) their implications for various smart home services, and (C) user experience. The analysis revealed that technology-based research (labeled as A in blue) has received the most attention. This focus revolves around the technological aspects of smart homes. Meanwhile, the focus on services (labeled as B, comprising concepts such as “Control and management,” “Systems,” “Energy,” and “IoT” related to the underlying technology and services derived from it) underscores the evolution toward exploring how these technologies can be harnessed to offer various services.

**Table 1:** Ranking of the identified concepts over the total period (2000 to the first half of 2023).

Concept	Count, n	Relevance percentage (n=3028; %)
Smart	2612	86
Use	1293	43
System	1161	38
IoT^a^	770	25
Devices	761	25
Data	760	25
Energy	756	25
Technologies	720	24
Activity	599	20
Model	551	18
Users	547	18
Sensor	537	18
Applications	509	17
Environment	496	16
Network	490	16
Security	408	13
Services	400	13
Learning	355	12
Information	322	11
Management	319	11
Different	319	11
Recognition	315	10
Present	297	10
Control	296	10
Monitoring	285	9
Consumption	283	9
Human	265	9

^a^IoT: Internet of Things.

Initially, smart home technology concentrated on managing home appliances through communication and control, leveraging the rapid advancements in electricity and information technology. There was also significant focus on how these technologies can be used to provide various services, highlighted by themes such as “Control and management,” “Systems,” “Energy,” and “IoT.” This indicates a shift from basic appliance management to broader service offerings through smart home technologies.

User-centered research themes in smart homes, while recognized as important, have limited attention focus compared with technological and service aspects. Discussions since the early 2000s have highlighted the need for a user-centered approach, focusing on specific groups such as the older adults and those with chronic conditions, under themes such as “Elderly” and “Active environment.” However, much of the research has been limited to usability studies that assess how these groups interact with and perceive smart home technologies, with less emphasis on understanding the broader spectrum of user needs and preferences. This focus remains a lower priority within smart home research, primarily observed through usability evaluations and pilot tests [64].

The results of the analysis provided insights into the current stage of the discipline, effectively addressing the third and fourth research questions. It identified that the optimal framework for smart home research lies in integrating technological innovation, service development, and user-centered experience to bridge the gaps between advancements and diverse user needs. Furthermore, the diachronic analysis of the past 20 years highlights the field’s evolution—from foundational technologies and energy management to the expansion of IoT applications—while emphasizing the growing importance of prioritizing user-centered needs.

### Deep Analysis by Time Series

In the previous section, thematic analysis was performed on the entire dataset of 1003 papers to provide a comprehensive overview of the entire period, and in this section, the period was temporally divided into 4 equal segments of 6 years for a more granular understanding of the thematic focus during each distinct period. By analyzing the literature from each period separately, the study aimed to understand the evolution of smart home research over time and identify any shifts or trends in research themes across different time frames.

**Figure 3:**
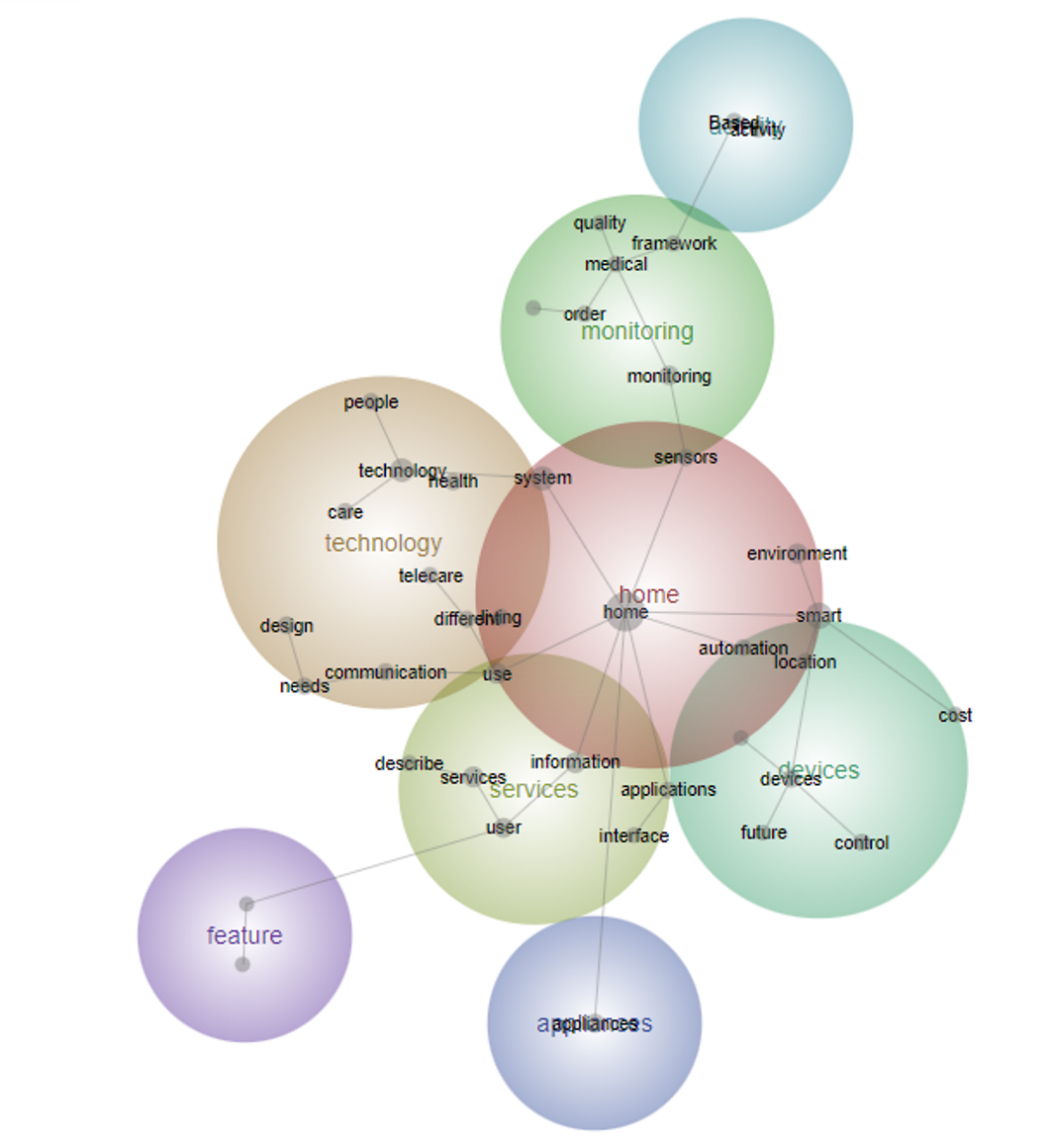
Concept map for period 1 (2000-2005).

The earliest period (2000-2005) represents the early literature phase, comprising 22 papers, which accounts for 2% of the total literature (n=1003), with the smallest number of text blocks (n=89). During this period, the key themes revolved around home, technology, services, and devices, with notable concepts including “Appliances,” “Information,” and “Services” (Figure 3). The literature review shows that this period was mainly dominated by technology-focused electrical equipment suppliers (eg, switches, sockets, and distribution boards) aiming to commercialize smart home solutions. The concept closely associated with technology was “System,” mentioned 23 times throughout the literature from this period (Table 2).

**Table 2:** Ranking of the identified concepts over period 1 (2000-2005).

Concept	Count, n	Relevance percentage (n=79; %)
Home	79	100
Smart	38	48
Technology	26	33
System	23	29
Services	20	25
Use	17	22
Environment	17	22
Appliances	17	22
Information	16	20
User	15	19
Monitoring	11	14
Devices	11	14
Design	10	13
Activity	10	13
Control	9	11
Needs	8	10
People	8	10

In the literature, smart homes are often described as advanced systems that leverage electricity and information technology to control household appliances. These systems encompass various functionalities, including but not limited to audio, video, home office equipment, telecommunication devices, intercom systems, security features, lighting controls, “HVAC” (heating, ventilation, and air conditioning) systems, and even lawn sprinklers [9].

During this period of smart home literature, the focus was primarily on basic communications technologies, often centered around services such as information provision and remote monitoring, particularly related to electricity usage. However, critiques emerged regarding the predominant emphasis on technological feasibility over user preferences and needs. User-centric considerations were not adequately integrated into the design and development processes, with minimal attention given to the user perspective and desires. Keywords related to users ranked relatively low in frequency (ie, the tenth highest among all concepts) compared with other concepts during this period (Table 2). Moreover, specific terms directly referencing users were notably absent. Instead, concepts such as “Needs” and “Environment” were more prevalent in discussions involving users. “Telehealth” emerged as a concept connected to the theme of “Technology,” with studies showing that phone calls can replace nursing visits. Critically, it was observed that the design and development of smart homes predominantly catered to the preferences and lifestyles of men, overlooking the needs of women who typically manage domestic tasks and are primary users of household technologies. Consequently, there was a recognized necessity to align smart home implementations with user needs, emphasizing the importance of user-oriented research. However, existing studies primarily remained theoretical, lacking empirical case studies to inform practical applications and implementations [7,9,29,30].

**Figure 4:**
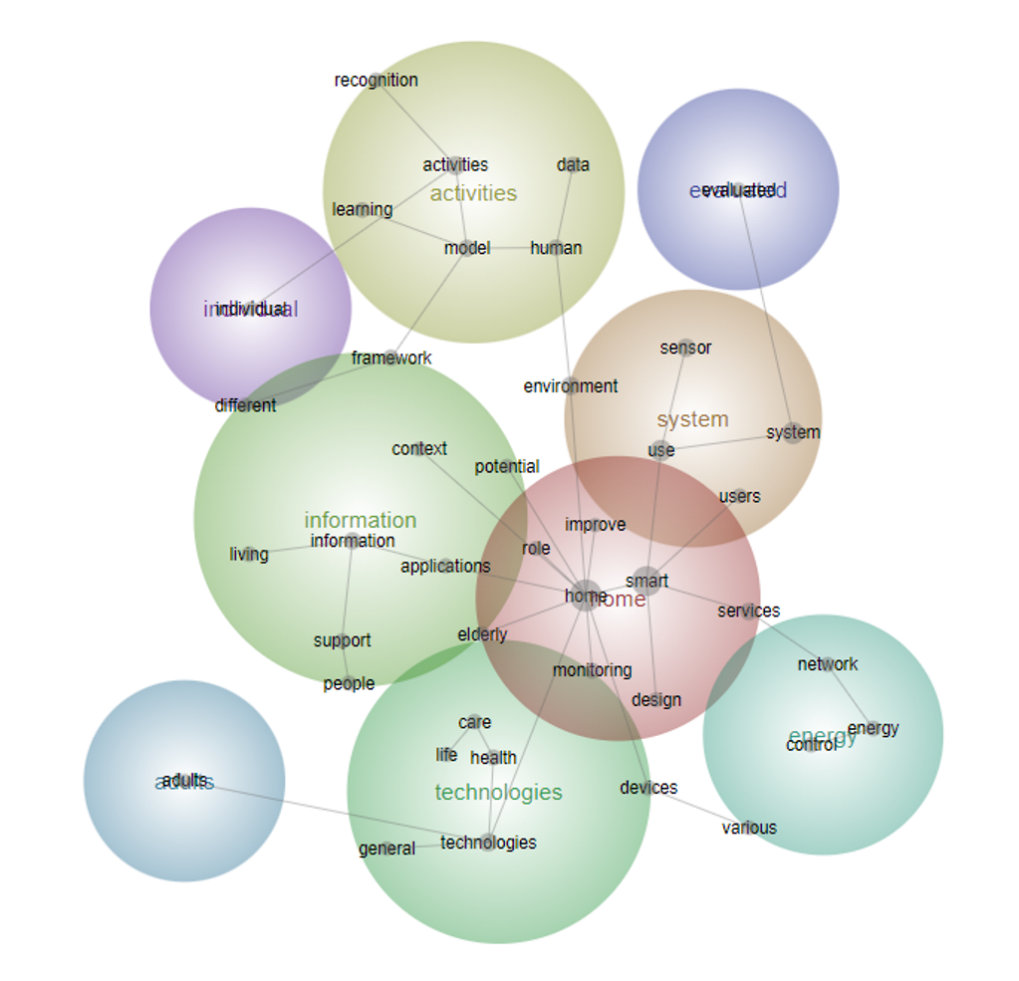
Concept map for period 2 (2006-2011).

During the second period (2006-2011), which comprised 81 papers, constituting 8% of the total literature (n=1003; 307 text blocks), the thematic focus shifted toward activity, sensors, and the older adults. While technology remained an important theme from the previous period, key concepts associated with it now included “Information,” “Health,” and “Monitoring” (Figure 4). The literature during this period witnessed the emergence of environments equipped with sensor technologies, enabling continuous monitoring capabilities. Situational awareness systems also gained prominence, facilitating communication network–enabled information dissemination to residents while collecting sensor-based data to deliver tailored services.

During the second period, the integration of technology with health-related applications became prominent, particularly in addressing the needs of the older adults and the individuals with disabilities [31]. Early research endeavors during this time aimed to empower these demographics with greater independence through smart home technologies [2].

Despite the growing recognition of the importance of end users in smart home design and implementation, user-related keywords, particularly those related to the older adults, emerged but remained relatively infrequent (Table 3). However, concepts such as “Device,” “Service,” and “Activity” began to emphasize a more user-centered approach, enhancing end user engagement and understanding. This involved using virtual environments, such as smart home simulations, to provide users with deeper insights into the setup and operation of smart devices and services [32-34].

**Table 3:** Ranking of the identified concepts over period 2 (2006-2011).

Concept	Count, n	Relevance percentage (n=192; %)
Home	192	100
Smart	166	86
System	101	53
Use	84	44
Activities	64	33
Technologies	52	27
Sensor	50	26
Environment	47	24
Services	43	22
Information	42	22
Model	41	21
Data	37	19
Framework	32	17
Human	28	15
Learning	26	14
Energy	25	13
Control	25	13
Health	23	12
Living	23	12
Monitoring	21	11
Support	21	11
Recognition	21	11
Care	19	10
Devices	19	10
Network	19	10
People	18	6
Applications	18	6
Elderly	17	6
Different	17	6
Users	16	5

**Figure 5:**
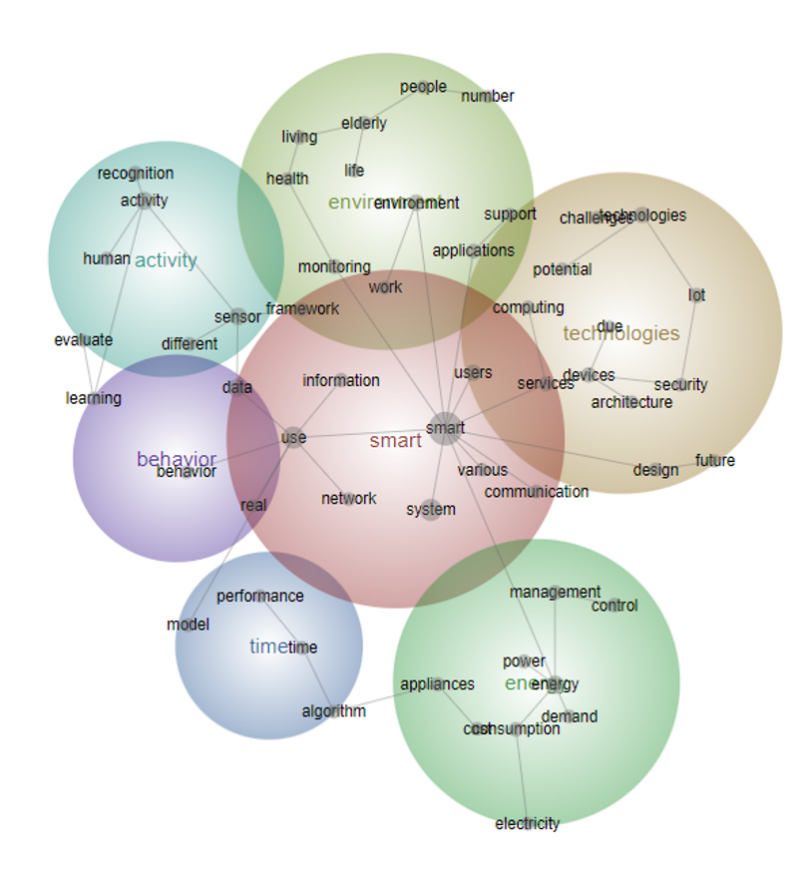
Concept map for period 3 (2012-2017).

The third period (2012-2017) comprised 278 papers, representing 28% of the total period (n=1003), with 1222 text blocks. Significant themes observed during this period included activity, technology, and energy, with users emerging as a major theme for the first time (Figure 5). Concepts related to technology, such as “Communication,” “Living,” and “Health,” were highly ranked during this time. In the literature of this period, a smart home was defined as a dwelling equipped with technology designed to anticipate and fulfill the needs of its occupants. There was a growing emphasis on the importance of well-managed technology and its integration with external connectivity to promote occupant comfort, convenience, and security. This connection to the outside world emphasizes the concept of an information home, where new information services are interactively connected, rather than simply automated [7,35].

**Figure 6:**
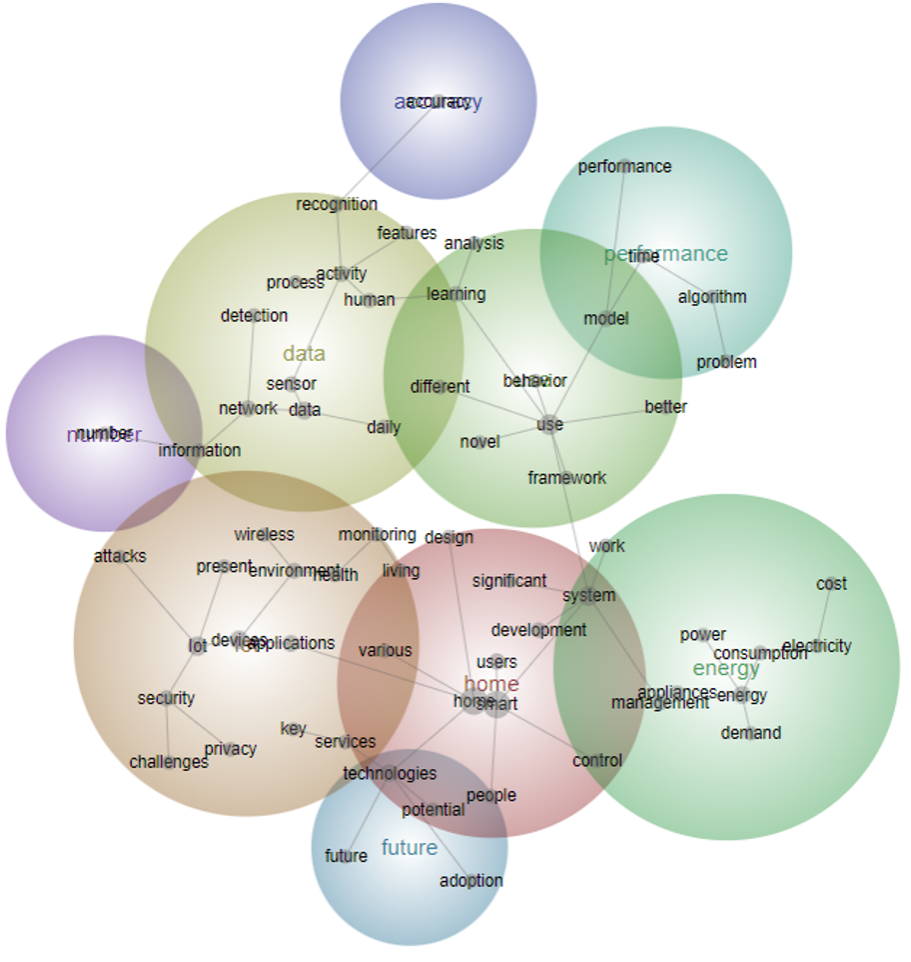
Concept map for period 4 (2018 to the first half of 2023).

During this period, “Energy” emerged as the fifth most important theme, with concepts linked to “Energy management,” “Consumption,” and “Control” (Figure 6 and Table 4). Research on users in smart homes delineates 3 main perspectives: functional, instrumental, and social. Functional perspectives focus on enhanced functionality and efficiency in smart homes. Instrumental perspectives emphasize energy savings and responsiveness to information and prices. Social perspectives focus on the adoption and use of technology in complex households [12,36]. Energy has mostly appeared in the literature revolving around services embedded in appliances that can monitor and control the power usage of appliances.

The study by Noury et al [65] emphasized the concept of “healthy smart homes” within the context of “Aging in Place,” expanding the definition of smart homes to include not only improvements in quality of life but also access to home care for the older adults and those with disabilities [37,38]. In developed countries with rapidly aging populations, there has been an increase in research to support applications that collect data from activities of daily living to help older adults with their daily lives through assisted living.

During this period, user emerged as the ninth most important concept, along with user-related concepts such as environment, behavior, and service, and there has been an increase in health-related research, especially focusing on the older adults and those with disabilities [38]. With the proliferation of smart home solutions and services, empirical studies have emerged that focus on users’ adoption intentions, utilization, and psychological aspects. For instance, research in China delved into the psychological aspects of end users [39], while another study applied the theory of planned behavior to investigate how user attitudes and subjective norms influence intentions to use smart home services, as well as psychological aspects [36]. In addition, there was a study on the emerging issue of single-person households, exploring strategies to enhance perceptions of social support in socially isolated environments [40].

**Table 4:** Ranking of the identified concepts over period 3 (2012-2017).

Concept	Count, n	Relevance percentage (n=739; %)
Smart	739	100
Use	349	47
System	343	46
Activity	205	28
Energy	200	27
Data	193	26
Sensor	175	24
Technologies	167	23
Users	154	21
Environment	148	20
IoT^a^	128	17
Devices	115	16
Recognition	105	14
Monitoring	98	13
Services	90	12
Applications	90	12
Control	90	12
Model	87	12
Different	84	11
Management	82	11
Time	80	11
Consumption	79	11
Power	74	10
Design	71	10
Living	69	9
Information	68	9
Elderly	67	9
Appliances	66	9
Security	65	9

^a^IoT: Internet of Things.

Finally, the fourth period (2018-2023) stands out as the most numerous, comprising 3030 text blocks, which account for 62% of the total literature (622/1003). This indicates a substantial surge in research activity during the last 6 years. Key themes during this period include “IoT,” “Data,” and “Energy,” highlighting the growing emphasis on interconnected technologies, data management, and energy efficiency. Emerging concepts such as “Models,” “Applications,” and “Adoption” reflect the evolving landscape of smart home research, with increasing attention on developing models, practical applications, and strategies for the widespread adoption of smart home technologies and services (Figure 6 and Table 5).

**Table 5:** Ranking of the identified concepts over period 4 (2018 to the first half of 2023).

Concept	Count, n	Relevance percentage (n=1443; %)
Home	1443	100
Smart	1442	100
Use	843	58
System	712	49
IoT^a^	644	45
Devices	580	40
Data	501	35
Energy	482	33
Technologies	466	32
Model	378	26
Network	362	25
Applications	361	25
Users	354	25
Security	329	23
Activity	321	22
Sensor	307	21
Environment	268	19
Learning	263	18
Development	237	16
Management	213	15
Different	212	15
Information	196	14
Performance	188	13
Recognition	182	13
Human	179	12
Control	172	12
Consumption	170	12
Privacy	170	12
Various	166	12
Detection	166	12
Services	160	11
Challenges	157	11
Monitoring	156	11

^a^IoT: Internet of Things.

The concepts of “Adoption,” “Application,” and “Potential” highlight technological advancements and the increasing integration of IoT within smart home systems. These advancements have led to continuous improvements in smart home services, allowing for seamless connectivity and control from anywhere at any time. A typical smart home system consists of various sensors and switches centralized around a main gateway, enabling users to access and manage their smart home devices through digital platforms such as smartphones or desktop personal computers [41].

In this period, concepts related to users are intricately linked with notions of privacy, security, and the overall environment. In addition, concepts related to usage, such as “Behavior” and “Learning,” underscore the importance of understanding user behavior, learning patterns, and the creation of models to enhance user experiences. The literature of this period includes empirical studies conducted in the United Kingdom, Finland, and China, which have provided valuable insights into users’ adoption and continuous usage of smart home technologies [42,43]. However, despite the growing interest and adoption, several barriers to widespread adaption have been identified [44]. These include mistrust and resistance, limited awareness of smart home capabilities, financial constraints, concerns regarding privacy and security, technology-related anxiety, and potential negative social impacts [45]. Moreover, recent literature has analyzed the literature of the last 20 years and categorized the evolution of smart homes into 3 generations, with the third generation characterized by IoT-based environments, emerging as the center of technological change and innovation. This generation is recognized for its transformative potential in urban and social contexts [14].

Health emerges as a consistent theme across all periods of smart home research, reflecting users’ desires for improved health care support and quality of life. From the early stages, there has been a focus on leveraging smart home technologies to effectively assist individuals with chronic conditions and aging populations, thereby enhancing their well-being and independence. The home environment offers a conducive setting for continuous health monitoring and disease prevention, aligning with users’ preferences for staying in familiar surroundings while receiving care.

Currently, numerous experimental and observational studies prioritize health care apps in smart homes to meet users’ needs for a healthy lifestyle through IoT and sensor technologies. However, there remains a gap between users’ expectations and the technologies and services currently available in smart homes. Therefore, users are particularly interested in tangible benefits such as practical energy services, energy conservation, cost savings, and environmental sustainability, which provide more direct and measurable impacts.

Health care remains the oldest and most fundamental user demand. While efforts through Smart Home Solutions [11] aim to enhance quality of life, they still fall short in bridging the gap between expectations and actual service delivery. Consequently, smart homes need to provide automated, context-aware, and personalized solutions that cater to diverse user needs and preferences, with a strong emphasis on health care–centric innovations [46,66].

This allowed us to address the fifth question concerning the primary themes of each period and provide a comprehensive answer. The SLR process facilitated a comprehensive examination of the sixth research question, identifying key challenges in the advancement of smart home technologies—such as user acceptance, technical limitations, privacy concerns, and the discrepancy between user expectations and technological capabilities—while offering insights into future directions in the field. At the same time, the study identifies emerging opportunities driven by advancements in IoT, health care support, sustainable environments, user-centered design, and the evolution toward ambient intelligence (AmI).

## Discussion

### Evolving From Home Automation to Ambient Home Care

**Figure 7:**
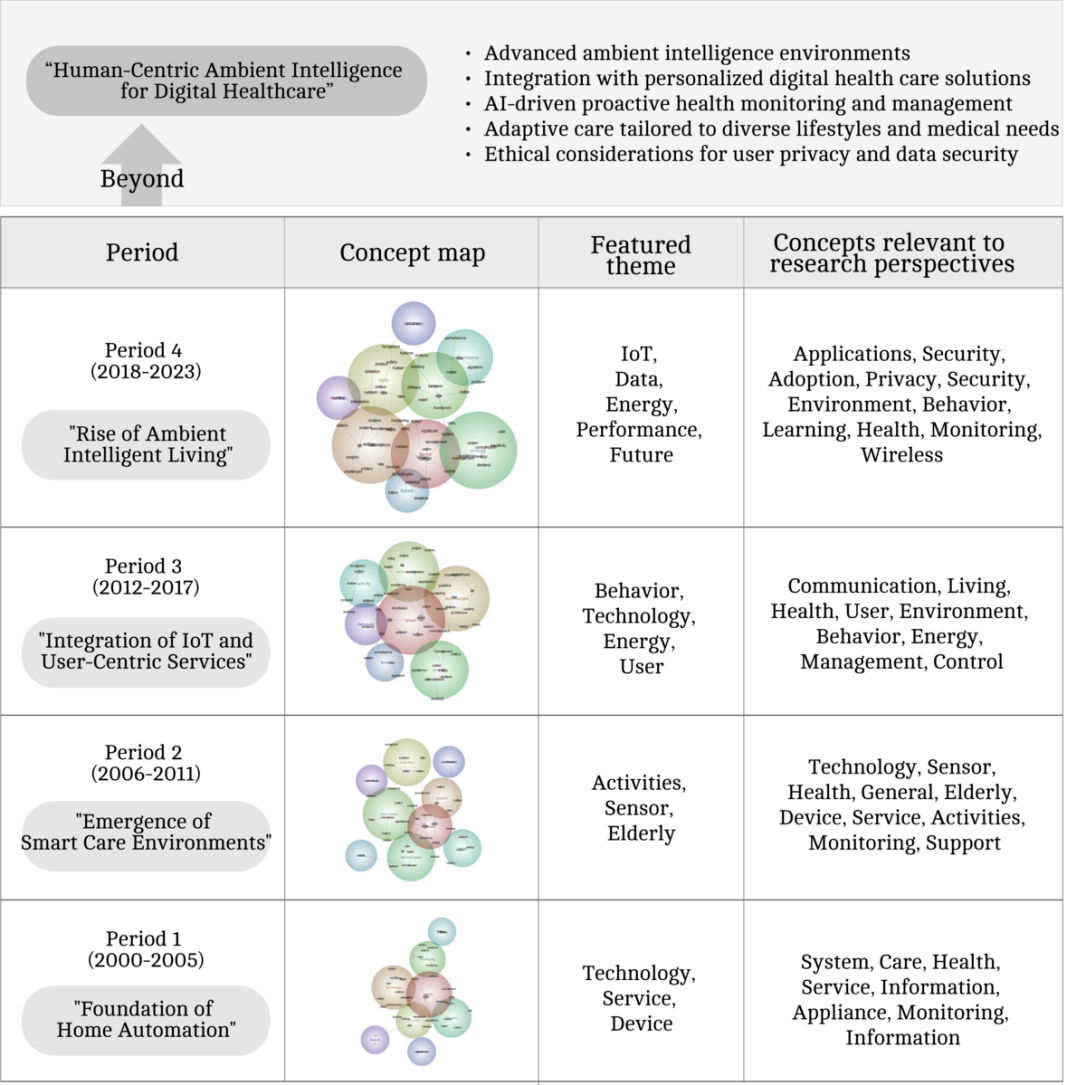
Concept map for overall period. AI: artificial intelligence; IoT: Internet of Things.

Early smart home systems focused on features such as monitoring electricity usage and automating basic tasks. However, as shown in Figure 7, the introduction of situational awareness systems and virtual environments has initiated a shift toward user-centric designs. These advancements have enabled the development of systems that support independence for older adults and individuals with disabilities while dynamically responding to user behaviors. Today, smart homes leverage AI and sensors to address diverse needs, including health care, privacy, and energy efficiency, significantly enhancing the quality of life for residents.

Despite these advancements, much of the existing research remains rooted in a technology-push approach, where the emphasis is placed on adoption and usability from the perspective of technology providers, often overlooking evolving user needs. For instance, early applications of smart home health care technologies primarily served caregivers by enabling patient monitoring, rather than directly benefiting patients themselves. The future of smart homes lies in the transition to AmI environments. These environments retain the technological innovations of the past but enhance them with user-centric principles, moving beyond the technology-push mindset to create spaces that intuitively adapt to residents’ needs and preferences.

The AmI environment represents a shift toward reducing the need for manual and explicit control by creating an environment that intuitively senses and responds to the user’s presence, preferences, and needs. These changes are clearly evident in the AmI home design, which is distinguished by its sensor-centric architecture. These homes are equipped with a variety of interconnected sensors and devices that collaborate seamlessly to create a responsive and adaptive living environment and improve activities of daily living [67].

To move forward, we need to think idealistically about the future. The core value proposition of a smart home lies in its ability to foster meaningful interactions with users and provide real value and convenience. The effectiveness of smart homes should be evaluated based on direct benefits such as increased convenience and improved quality of life, but these user-centric values are often overshadowed by the practical difficulties of adoption [68]. Likewise, financial constraints, such as high device and installation costs, limit accessibility, especially for low- and middle-income households [69].

By overcoming these barriers, smart homes have the potential to become truly inclusive and life-changing spaces that meet the needs of diverse user groups. In the ever-evolving smart living environment, this field is about to take a revolutionary leap forward, filled with technologies that not only simplify but also deeply enrich life [34,68-70].

This discussion aligns with the abstract’s emphasis on overcoming the limitations of technology-centric approaches and highlights the importance of creating smart homes that prioritize user satisfaction, inclusivity, quality of life, and enhancing user satisfaction and quality of life, which are fundamental in improving the quality of life.

### The Evolution of Smart Homes Toward Improved Health and Quality of Life

This study analyzed 5983 smart home papers from the past 20 years, using thematic analysis techniques to discern trends and evolutionary patterns across technology, service, and user perspectives. The research identified a significant increase in smart home literature over the last 6 years, with prominent journals such as *Sensors* and *IEEE* featuring heavily cited works, reflecting a strong emphasis on technology. A notable shift was observed from basic monitoring and remote-control functions to more user-centric services such as energy management and health care, facilitated by advancements in sensors, IoT, and data technologies.

In particular, health care in the smart home is the first and earliest expanded research direction [14,71], indicating that the ultimate purpose of the smart home will evolve toward facilitating independent living and improving the quality of life. This is achieved through remotely managed services that provide tailored solutions such as chronic disease management, fall detection and prevention, automated medication adherence, mental health support, and improved accessibility for individuals with disabilities, effectively catering to the diverse needs of its users [72].

The study offers a comprehensive scholarly review, helping researchers understand the field’s development and guiding future research directions. Currently, practical energy services and environmental sustainability represent the areas where the gap between user expectations and available technology is smallest and benefits are most evident within smart home environments. Therefore, addressing health care, which remains the oldest and most fundamental user demand, involves implementing various forms of remote monitoring and telemedicine services to effectively meet these needs. These insights provide valuable guidance for shaping future academic and commercial strategies in smart home technology. However, limitations include the use of only paper titles and abstracts for analysis, potentially missing details from full texts and excluding nonacademic perspectives. Future research could improve by incorporating full-text analyses and industry stakeholder insights.

In summary, the analysis provides a valuable foundation for future smart home research and commercialization, emphasizing the importance of addressing user needs in academic and industry applications, and has managerial implications for future smart home products and services.

## Appendix

Multimedia Appendix 1 PRISMA (Preferred Reporting Items for Systematic Reviews and Meta-Analyses) 2020 checklist.

Multimedia Appendix 2 Graph illustrating the accelerating trend in academic interest, which has shown steady growth since 2018.

Multimedia Appendix 3 Graph showing a list of the top impactful journals from 2000 to the first half of 2023.

## Abbreviations

AI: artificial intelligence
AmI: ambient intelligence
B2B: business-to-business
IoT: Internet of Things
PRISMA: Preferred Reporting Items for Systematic reviews and Meta-Analyses
RQ: research question
SLR: systematic literature review
