# Supplementary figures and images for: Smart Home Advancements for Health Care and Beyond: Systematic Review of Two Decades of User-Centric Innovation
Source: J Med Internet Res. 2025 May 20;27:e62793. doi: 10.2196/62793 (PMC12134694; doi:10.2196/62793)

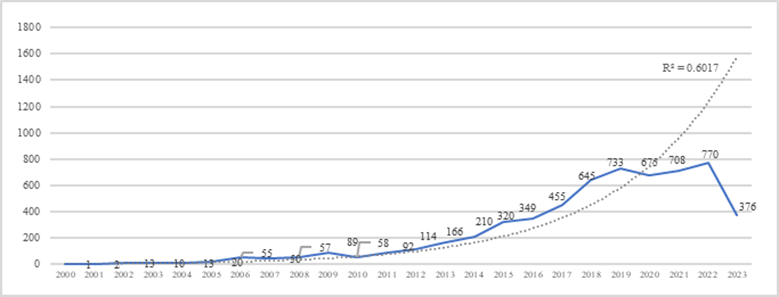

Supplement: Multimedia Appendix 2 [file jmir_v27i1e62793_app2.png]

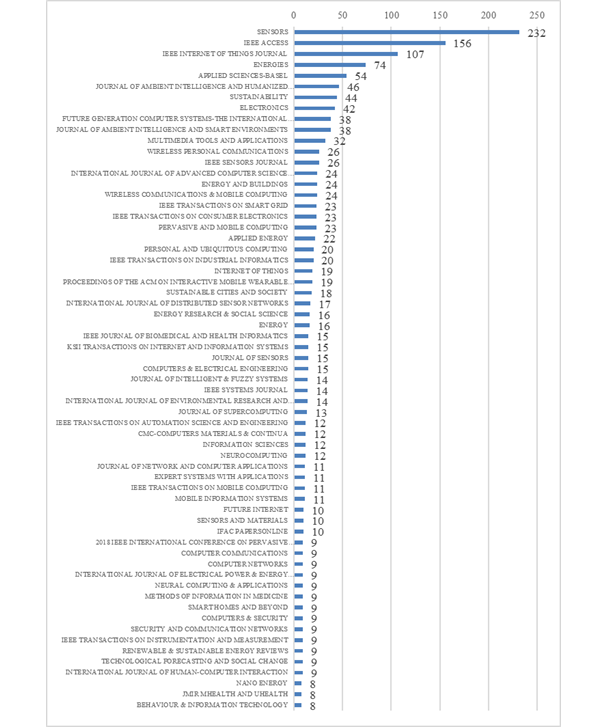

Supplement: Multimedia Appendix 3 [file jmir_v27i1e62793_app3.png]
